# Application of Nanomaterials and Related Drug Delivery Systems in Autophagy

**DOI:** 10.3390/molecules29153513

**Published:** 2024-07-26

**Authors:** Ling Mei, Kai Liao, Haiyan Chen, Yifan Zhang, Zihan Zhang, Qiangwei Li, Man Li

**Affiliations:** 1 Engineering Research Center for Pharmaceuticals and Equipment of Sichuan Province, Sichuan Industrial Institute of Antibiotics, School of Pharmacy, Chengdu University, Chengdu 610106, China; meiling@cdu.edu.cn (L.M.);; 2 Key Laboratory of Drug-Targeting and Drug Delivery System of the Education Ministry and Sichuan Province, West China School of Pharmacy, Sichuan University, Chengdu 610041, China; 3 Sichuan Engineering Laboratory for Plant-Sourced Drug, Sichuan Research Center for Drug Precision Industrial Technology, Sichuan University, Chengdu 610041, China

**Keywords:** nanoparticle, autophagy, tumor targeted, drug delivery, nanomaterials

## Abstract

Autophagy, a lysosomal self-degradation pathway, plays a critical role in cellular homeostasis by degrading endogenous damaged organelles and protein aggregates into recyclable biological molecules. Additionally, it detoxifies extracellular toxic substances, including drugs and toxic materials, thereby preserving the stability of the intracellular environment. The swift progression of nanotechnology has led to an increased focus on understanding the relationship between nanomaterials and autophagy. The effects of various nanomaterials and nano drug delivery systems on autophagy and their biological functions have been preliminarily assessed, revealing that modulation of intracellular autophagy levels by these agents represents a novel cellular response mechanism. Notably, autophagy regulation based on nanomaterials or nano drug delivery systems for a range of diseases is currently the subject of extensive research. Given the close association between autophagy levels and tumors, the regulation of autophagy has emerged as a highly active area of research in the development of innovative tumor therapies. This review synthesizes the current understanding of the application of nanomaterials or nano drug delivery systems on autophagy and their potential biological functions, suggesting a new avenue for nanomaterial-based autophagy regulation.

## 1. Introduction

**Figure 1:**
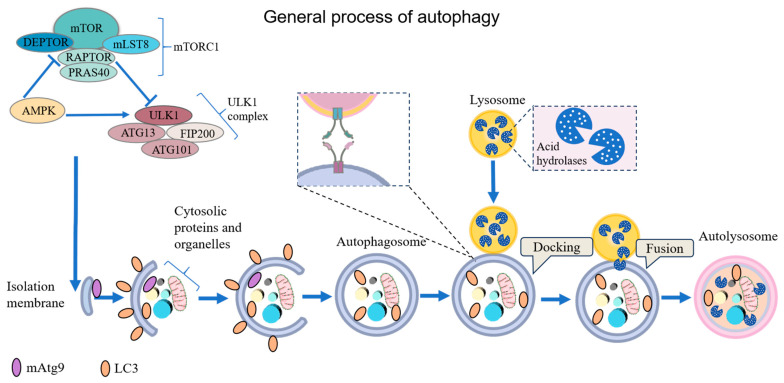
The general process of autophagy and the regulatory network of related signaling molecules.

Autophagy is a highly conserved process of self-digestion and catabolism, in which non-functional or excess cellular components are engulfed by autophagosomes and degraded by lysosomes. The general process includes activation of autophagy-related signaling pathways, formation of autophagy precursors, formation of autophagosome containing substances to be cleaned, fusion of autophagosome and lysosome, final degradation, and reuse of substances (Figure 1) [1]. Autophagy is a mechanism widely used by all eukaryotic cells to cope with internal and external adverse factors. The sustained process of autophagy dysregulation has been found in various diseases, including neurodegenerative diseases, inflammatory diseases, and tumors. Due to the lysosome-dependent material degradation pathway, autophagy plays an important role in cleaning up the unwanted proteins, dysfunctional organelles, and exogenous invading substances such as bacteria and drugs [2].

The integration of nanotechnology into the study of autophagy has given rise to a new class of diagnostic tools known as nanomaterial-based autophagy probes. These innovative probes leverage the unique physical and chemical properties of nanomaterials to provide enhanced detection and quantification of autophagy activity within cells. Nanomaterial-based autophagy probes offer several advantages over traditional methods [3]. Their small size allows for deep tissue penetration and cellular uptake, enabling researchers to monitor autophagy processes in real time with high spatial resolution. Additionally, the surface of these nanomaterials can be functionalized with specific molecular ligands that target autophagy-related proteins or organelles, thereby increasing the specificity and sensitivity of the detection [4]. One of the most promising applications of these probes is in fluorescence imaging, where nanomaterials with photoluminescent properties can be used to visualize autophagic structures such as autophagosomes and autolysosomes. The fluorescence signal emitted by these probes can be correlated with the level of autophagy, providing a quantitative measure of the process. Moreover, the development of multifunctional nanomaterial probes allows for the simultaneous detection of autophagy and other cellular processes. For example, probes can be designed to report on both autophagy and apoptosis, providing insights into the complex interplay between these two pathways in disease progression [5].

Nanotechnology has advanced the field of autophagy research and therapy by introducing nanomaterials with the unique ability to either induce or inhibit autophagy, depending on the desired outcome for a specific disease condition. Certain nanomaterials are designed to stimulate autophagy, which can be beneficial in scenarios where the clearance of abnormal proteins or damaged organelles is necessary. In conditions such as atherosclerosis, where plaque buildup can be partly attributed to the accumulation of waste materials within cells, the use of autophagy-inducing nanomaterials may help in the degradation and removal of these waste products, reducing the plaque burden [6]. At the same time, autophagy has a close relationship with a variety of human tumors [7], which suggests that autophagy is an entry point for tumor therapy. The level of autophagy within tumor cells varies with different stages of tumor growth and the spatial location of tumor cells in the tumor tissue. Autophagy usually plays a crucial role with a dual effect of promoting and inhibiting the tumor growth. There is a parabolic relationship between autophagy level and tumor cell activity: when the autophagy level is in the “equilibrium range”, tumor cells can benefit from autophagy and maintain the optimal homeostasis. When the autophagy levels deviate from this range, the tumor growth will be inhibited. When the autophagy level deviates further from this interval, that is, when the autophagy level is too high or too low, the degree of tumor growth inhibition is also greater. There is an autophagy balance in the tumor microenvironment, and once this balance is disrupted, the process of tumor occurrence and development will be disrupted. Therefore, specific regulation of autophagy balance in the tumor microenvironment is a promising tumor treatment strategy, but it is also a research challenge [8]. However, it is difficult to obtain the ideal therapeutic effect due to the non-selective distribution of autophagy regulators in vivo. Traditional autophagy modulators often require frequent and high-dose administration to exert their autophagic interference function. The toxic side effects brought about by this multiple, high-dose administration method limit their application in clinical tumor treatment. In recent decades, the development of tumor-targeting drug delivery systems has highlighted its advantages in tumor treatment, and this is expected to be one of the breakthroughs to overcome tumors [9,10]. Due to the rapid growth of tumor tissues, tumor neovascularization and lymphatic vessels are usually incomplete, which enhances the permeability and retention effect (EPR effect) on nanoparticles of a certain size [11]. Based on the EPR effect, nano drug delivery systems can passively target the tumor site, so as to efficiently deliver drugs to the tumor [12]. At present, a variety of nanocarriers based drugs are on the market, such as paclitaxel albumin nanoparticles, doxorubicin liposomes, and paclitaxel micelles, and have achieved certain therapeutic effects [13,14]. Compared with free drugs, the nano drug delivery system has multiple advantages, including protecting drugs from degradation in the process of systemic circulation, changing the pharmacokinetics and tissue distribution behavior of drugs in vivo, ensuring the selective distribution of drugs in the body, and improving the accumulation of drugs at the tumor site [15]. The research on the regulation of the tumor autophagy level based on nano drug delivery systems is also getting more and more attention. This review briefly summarizes the current research of nano drug delivery systems based on autophagy regulation.

## 2. Nanomaterial-Based Probes for Monitoring Autophagy

Autophagy is a self-stabilizing mechanism of cells, and its activation state fluctuates rapidly with the change in cell environment. Due to its close association with various diseases, autophagy is a diagnostic and therapeutic target for certain diseases [16]. The real-time autophagy monitoring can indirectly help to understand the state of cells and related diseases. At present, the main methods for autophagy monitoring are transmission electron microscopy, Western blot, and confocal observation of the fluorescent autophagy protein mRFP-EGFP-LC3. These methods require pretreatment of cells, which may not only complicate the operation steps, but also affect the activity of cells. And it is difficult to accurately reflect the state of cells in real time as well. Therefore, it has become one of the research hotspots of autophagy drug delivery systems to find a suitable way to monitor the autophagy level of living cells. In recent years, researchers have attempted to develop many excellent probes for detecting autophagy [17,18]. Nanostructured probes, due to their unique structural and functional characteristics, are an interesting and promising method for in vivo biological imaging, mainly including inorganic hybrid nanomaterials and self-assembled peptide polymer nanoparticles. This includes nanoprobes constructed in situ based on the “in vivo self-assembly” strategy and responsive self-assembled nanoprobes [19]. The increase in reactive oxygen species (ROS) is a marker for certain pathological conditions, such as hypoxia, ischemia/reperfusion, and tumors, where mitochondrial ROS is upregulated and serves as an important signal for autophagy induction. Conversely, autophagy can reduce oxidative damage by clearing oxidative substances. The process involves the formation of autophagosomes that engulf damaged organelles and proteins, which are then delivered to lysosomes for degradation. This helps to reduce the accumulation of oxidatively damaged macromolecules and mitigates the potential for further oxidative damage. Therefore, there is a positive correlation between intracellular mitochondrial ROS and autophagy levels. Some metal nanoparticles exhibit unique optical properties in the visible and near-infrared regions, which enables detection via optical methods such as plasma resonance scattering (PRS) spectroscopy. Based on this, Chen et al. developed gold@silver nanorods with core–shell structures (Au@AgNRs). Due to the sensitivity to changes in the refractive index, it served as a probe to track autophagy processes by monitoring intracellular O_2_•− in real time [20]. The absorption peak in the PRS spectrum of Au@AgNRs underwent a red shift from 580 nm to 675 nm, and the color of the scattered light changed from yellow to red. Autophagy levels could be reflected by calculating the intracellular O_2_•− concentration. However, autophagy detection via measuring intracellular ROS also has some drawbacks. ROS may also be induced by tumor damage or other pathological conditions, which did not show a linear relationship with autophagy. Therefore, this method is currently only applicable for the qualitative evaluation of autophagy. Nowadays, nanoprobes based on tumor microenvironment response are receiving increasing attention. ATG4 protease is an indispensable enzyme in the autophagy process. ATG4 is responsible for the processing of ATG8-family proteins, which are essential for the formation of the autophagosome. ATG4 cleaves the C-terminus of ATG8, generating a form that can be conjugated to the autophagosome membrane. Thus, it could be used as a potential biomarker for autophagy [21]. Choi et al. developed a peptide-conjugated polymer nanoparticle probe for real-time visualization of ATG4 activity in living cells [22]. Modification of peptides is also possible with nanoprobes, the fluorescent dye FITC, and a quencher (STFGFSGKRRRR) to form FITC peptide conjugates, which could ultimately self-assemble into stable nanoparticles (TFG-HGC) through fluorescence energy transfer (FRET) effect. Meanwhile, loading a lysosomal staining dye (Lysolite Red) into the hydrophobic core of nanoparticles could distinguish between ATG4 enzymatic reaction and non-specific lysosomal degradation. Min Li et al. developed an autophagy probe based on fluorescence resonance energy transfer (FRET). The fluorescent dyes CFP and YFP were bound to both ends of the autophagy marker protein LC3B to form a fusion protein with FRET effect (named FRET-LC3B) [23]. The fusion protein can be used to detect ATG4B or ATG4B enzyme activity [24]. Supramolecular self-assembled nanoparticles have been employed to show intracellular dynamic processes involving lysosomes and autophagy as well. Datta et al. prepared a peptide-based, fluorescent sensor, HCFP, for tracking the autophagic flux, which was due to the pH fluctuations [25]. Enhanced red fluorescence and reduced green fluorescence indicated increased autophagic flux, and the fluorescence changes could be real-time monitored in confocal laser scanning microscope (CLSM) images. Since the specific substrate of ATG4B is the “TFG” sequence, researchers have designed a series of autophagy detection probes using TFG peptides. Hao’s group modified the photosensitizer Purpurin-18 on the surface of PAMAM (Poly(amidoamine), which is a type of dendritic polymer) through the polypeptide GKGSFGFTG, and constructed an autophagy-level-monitoring nanoprobe. When ATG4B enzyme activity was enhanced, the GKGSFGFTG polypeptide could be cut off, and the released Purpurin-18 could be clustered under its own hydrophobic effect, emitting strong fluorescence. This probe can realize “real-time monitoring” of the autophagy level in living mice, so as to guide the timing of chemotherapy drug administration. Similarly, some researchers introduced the water-soluble substrate amino acid chain of ATG4, the key protease downstream of the autophagy pathway, into the fluorescent system of a quinolinonitrile mother, and developed an enzyme-activated AIE (Aggregation-Induced Emission) autophagy detection probe, QM-GFTN [26]. Using enzyme digestion to regulate the response process of fluorescence to autophagy established a positive correlation between fluorescence intensity and autophagy, and achieved a simple, specific, and highly sensitive fluorescence-activated autophagy detection [27]. The peptide-based enzyme-activated probe integrates real-time sensing and a highly specific response, and has made a major breakthrough in detecting autophagy in a variety of autophagic cells, animal tissues, and even human pathological tissues. Recently, under the leadership of Howard H Chen, an autophagy detection nanoparticle was developed, which could image autophagy and measure its flux non-invasively in vivo [28]. These nanoparticles are modified with peptides rich in arginine that can be cut by cathepsin. It has the similar function as the near-infrared fluorescent pigment Cy5.5, and can promote the uptake of nanoparticles by early autophagosomes. The autophagy flux can be measured by magnetic resonance or near-infrared fluorescence imaging of intravenous iron oxide nanoparticles. However, these methods also face challenges, including the qualitative limitations of ROS-based autophagy detection, potential cross-reactivity, and the complexity and cost associated with the synthesis and application of these nanoprobes. The future of this field lies in improving the specificity and sensitivity of these probes, expanding their applications, simplifying the technology, and translating these tools into clinical practice for more reliable and accessible autophagy detection.

## 3. Bioactive Nanomaterials for Autophagy Regulation

Autophagy can be activated in response to a variety of intracellular and extracellular stimuli, including but not limited to starvation, hypoxia, chemotherapy, radiotherapy, photothermal therapy, photoacoustic therapy, mechanical injury, and so on. It was found that some nanoparticles could induce autophagy to some extent, such as fullerenes, carbon quantum dots, graphene, and gold nanoparticles [29]. Table 1 summarizes some typical nanomaterials, which have been found to regulate autophagy. Gu’s group has developed D-shaped peptide dendrimer materials (D-PNs), which could activate autophagy without the help of chemotherapy drugs [30]. It can be further used to improve antitumor drug efficiency and reverse drug resistance. Nanoparticles can activate autophagy signaling pathways via different mechanisms, for example via stress induced by nanomaterials: The interaction between nanomaterials and cell surface receptors can activate intracellular signal cascades and induce the production of reactive oxygen species (ROS) in cells. When it is out of balance with the antioxidant defense system, it triggers the oxidative stress effect of cells [31], which is one of the important reasons for inducing autophagy [32]. Oxidative stress of cells caused by nanomaterials leads to lipid peroxidation and enhanced permeability of mitochondrial membranes, decreased mitochondrial membrane potential (MMP) and adenosine triphosphate (ATP), leading to swelling of mitochondria and release of cytochrome C from mitochondrial membranes [33]. Damaged mitochondria maintain cytoplasmic homeostasis by activating autophagy to remove dysfunctional organelles. Nanoparticles with iron cores and gold shells (Fe@Au) were proven to cause an irreversible membrane-potential loss in the mitochondria of cancer cells and ROS accumulation, and to finally inhibit hNOK cell proliferation through mitochondria-mediated autophagy [34]. At the same time, endoplasmic reticulum stress (ERS) is also one of the main ways to cause autophagy. Nanomaterials can induce the accumulation of unfolded or misfolded proteins in the endoplasmic reticulum after entering cells, causing ERS. Park et al. found that magnetic iron oxide nanoparticles (M-FeNPs) could induce autophagy through mitochondrial damage and endoplasmic reticulum stress on mouse peritoneal giant cells (RAW 264.7) [35]. When cells underwent a 24 h exposure to M-FeNPs following pretreatment with the autophagy inhibitor bafilomycin A1, significant cellular changes were observed. The integrity of the plasma membrane was compromised, resulting in the disappearance of cytoplasmic contents, and there was a notable rise in the incidence of apoptosis. The ingestion of nanoparticles via endocytosis presents a challenge for lysosomal degradation, frequently resulting in lysosomal impairment and subsequent organelle dysfunction. Upon entry into human trophoblast cells, TiO_2_ nanoparticles predominantly localized within lysosomes, impairing their degradative capabilities and causing leakage of these particles into the cytoplasm. The nanoparticles’ interaction with cytoplasmic proteins led to protein destabilization, triggering endoplasmic reticulum stress and mitochondrial dysfunction, which in turn promoted the clearance of damaged mitochondria through autophagy, as referenced in previous studies [36]. Similarly, in macrophages, the aggregation of single-walled carbon nanotubes and graphene oxide within lysosomes was found to compromise lysosomal membrane integrity and hinder the breakdown of autophagic substrates [37]. Gold nanoparticles have also been shown to induce lysosomal swelling, elevate lysosomal pH, and diminish the proteolytic activity of lysosomal enzymes, leading to an accumulation of autophagic substrates and the induction of cellular autophagy [38]. The damage to lysosomes by nanoparticles can also lead to secondary effects, such as inducing oxidative stress within cells. Overexposure to reactive oxygen species can impair autophagy, resulting in the buildup of damaged organelles, including mitochondria. This accumulation can trigger oxidative stress, inflammation, and DNA damage, potentially culminating in autophagic dysfunction that may precede apoptosis or cell death through autophagy, thereby diminishing the cell survival [39]. In research conducted by Mittal and colleagues, it was discovered that graphite carbon nanofibers could inflict damage upon the lysosomal structures and cytoskeleton of lung cells, obstructing the flow of autophagy and leading to an accumulation of autophagosomes. This accumulation was linked to an increase in intracellular reactive oxygen species (ROS), which in turn induced apoptosis [40]. Cell autophagy induced by nanomaterials may damage cells on the one hand, and may also protect cells on the other hand. Huang et al. found that oxidative stress could induce DNA damage and activate poly(ADP-ribose) polymerase 1 (PARP-1). PARP-1 induced autophagy through the AMPK/mTOR pathway, and the occurrence of autophagy improved the cell survival rate [41]. When the autophagy-related genes ATG5 or ATG7 were knocked out, the cell death rate increased significantly. Zhang et al.’s research showed that TiO_2_ nanomaterials could induce low-level autophagy by activating the AMPK/mTOR signaling pathway, and reduced TiO_2_ induced cell death [42]. At present, protective autophagy induced by nanomaterials is widely used in the treatment of some diseases [43,44]. Nanographene oxide (GO) was found to enhance the fusion of autophagosomes and lysosomes, and protect nerve cells against prion protein (PrP (106–126))-mediated apoptosis [45]. In conditions like Alzheimer’s or Parkinson’s disease, where the accumulation of misfolded proteins is a problem, nanoparticles could be engineered to target these proteins. The induced autophagy could help in the degradation and recycling of these proteins, thus reducing their toxic accumulation and slowing disease progression [46]. In wound healing or after surgery, nanoparticles could be used to stimulate autophagy in local cells [47]. This could promote the clearance of dead or damaged cells and debris, facilitating a cleaner environment for tissue regeneration and faster recovery. In summary, while bioactive nanomaterials hold promise for the regulation of autophagy and the treatment of various diseases, they also pose potential risks due to their interactions with cellular organelles. The balance between the damaging and protective effects of induced autophagy by nanomaterials is a critical consideration in their design and application for therapeutic purposes. Future research should focus on understanding these complex interactions to optimize the beneficial effects of nanomaterial-induced autophagy while minimizing potential harm.

## 4. Nano Drug Delivery Systems (DDS) for Autophagy Regulation

**Figure 2:**
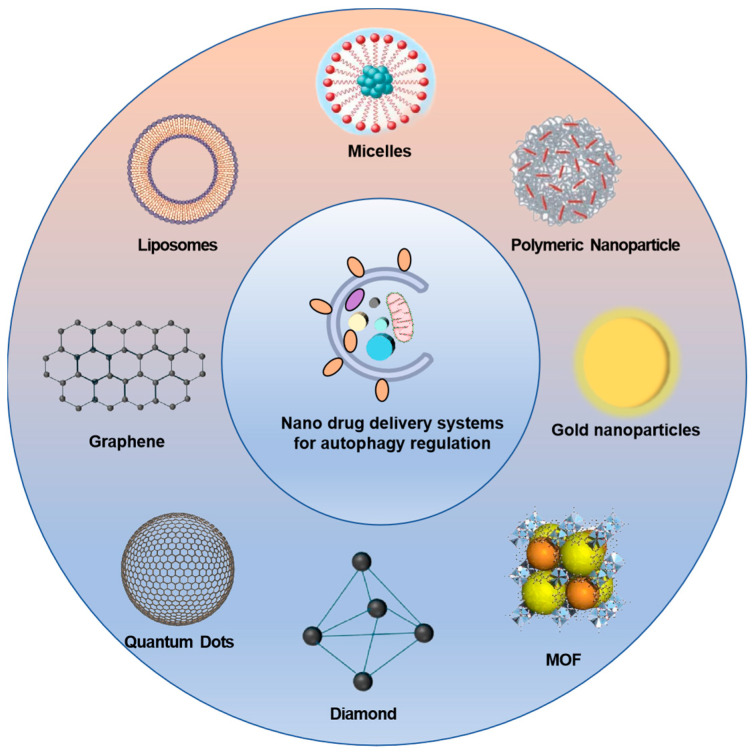
Nano drug delivery systems for autophagy regulation.

Autophagy is a double-edged sword, which can not only promote tumor development, but also inhibit tumor growth under different conditions [58]. Usually, the overly activated or inhibited state of autophagy can induce cell death of tumor and achieve anti-tumor effects. In tumor therapy, nanoparticles can be used alone or as carriers for the delivery of different types of molecules, such as chemotherapeutics, autophagy regulators, or antibodies, in order to enhance treatment effectiveness via modulation of autophagy. The main nano drug delivery systems include organic nanoparticles (lipid-based nanoparticles and polymer nanoparticles) and inorganic nanoparticles (Figure 2). Lipid nanoparticles (LNPs) stand out as a prominent drug delivery platform, offering a multitude of benefits such as ease of formulation, inherent self-assembly capabilities, compatibility with biological systems, enhanced bioavailability, and the capacity to encapsulate substantial drug payloads [59]. Their physicochemical attributes can be finely tuned to modulate their interaction with biological systems, which has made them a prevalent choice in nanomedicines approved by regulatory bodies like the FDA. Polymeric nanoparticles represent another class of drug carriers that can be crafted from either naturally occurring or synthetic polymers, enabling a wide array of structural and functional configurations. These particles are particularly well suited for the co-delivery of therapeutic agents, accommodating both hydrophobic and hydrophilic drugs. They can encapsulate a diverse range of molecular weights, from small molecules to larger entities such as biologics, proteins, and vaccines. By meticulously adjusting parameters such as the nanoparticles’ composition, stability, reactivity, and surface charge, along with those of the encapsulated drugs, the efficiency of drug loading and the release profile can be precisely managed [60]. Inorganic nanoparticles encompass a broad spectrum, including metallic elements, metal oxides, carbon-based materials, and magnetic nanoparticles, with superparamagnetic iron oxide nanoparticles (SPIONs) being a notable example [61]. Among these, mesoporous silica nanoparticles (MSNs) have emerged as a preferred option due to their well-defined pore structure, ease of modification, biocompatibility, substantial surface area, ample pore volume, and biodegradable nature.

### 4.1. Nano DDSs for Autophagy Induction

The commonly used treatment methods for inducing autophagy include chemotherapy drugs, photosensitizers (photothermal therapy and photodynamic therapy), and some other drugs. Beclin-1 is a typical specific signal peptide for autophagy induction with a certain potential for tumor inhibition [62]. However, the instability and non-selective distribution in vivo limit its application. Yiyun developed a melanin-like nanoparticle decorated with an autophagy-inducing peptide beclin-1 for efficient targeted photothermal therapy, which successfully induced autophagic death of MDA-MB-231 cells, and enhanced photothermal therapy [63]. Wang et al. modified the autophagy-activating peptide beclin-1 onto pH-sensitive polymers (Ps), and a pH-responsive nano micelle p-bec1 was developed. When p-bec1 reached the MCF7 cells, protonation of the polymerized β-amino ester and micelle disintegration caused the peptide beclin-1 to enter into MCF7 cells under positive electricity, leading to autophagic death [64]. Similarly, Wang et al. adopted the strategy of combining autophagy overactivation and immunogenic death to build a “core–shell” structure nano drug delivery system leading to a double response of reduced glutathione and autophagy levels in CT26 cells, which triggered autophagic cell death, cooperated with chemicals to kill tumors, and promoted the antigen-processing and -presentation process of dead cells [65]. To regulate autophagy more accurately, Professor Zhang et al. delivered high-dose autophagy inducers using new nanocarriers targeting transferrin receptors. They could induce excessive autophagy of tumor cells and make cells unable to maintain homeostasis. Later, the combination of cytotoxic chemotherapy drugs aggravated the damage of intracellular organelles, leading to the excessive consumption of key intracellular mechanisms required to maintain cell viability, thus leading to autophagic cell death [66]. In that study, autophagic cell death was induced by regulating the concentration, administration time, and order of autophagy-activator-loaded nanoparticles combined with chemotherapy drugs.

### 4.2. Nano DDS for Autophagy Inhibition

Autophagy, as a self-homeostasis mechanism of cells, can help cells digest damaged components, recycle degraded products, and provide energy for cells. When chemical drugs are used to kill tumors, autophagy can help tumor cells clear the components damaged the drugs, maintain tumor survival, and reduce the efficacy of chemical drugs. Therefore, co-delivery of chemotherapeutic drugs and autophagy inhibitors has great potential to achieve a synergistic tumor killing effect. Related research is also gradually growing. Due to the non-selective distribution of autophagy inhibitors in vivo, the design of nano drug delivery systems for co-delivery is an important research purpose. Yang et al. encapsulated hydroxychloroquine, a classical autophagy inhibitor, into the aqueous phase of liposomes by a pH gradient method, modified tumor targeting peptide peptides on the surface of liposomes, constructed a liposome TR-HCQ/Lip, and realized the tumor-targeted delivery of autophagy inhibitors. In vivo anti-tumor experiments in 4T1 bearing mice have confirmed that TR-HCQ/Lip can more effectively reduce the gastrointestinal side effects of hydroxychloroquine and improve the anti-tumor effect of doxorubicin [67]. Xuhui et al. encapsulated the autophagy inhibitor wortmannin in brain-targeted liposomes, which can effectively inhibit autophagy in mouse brain capillary endothelial cells, thus inhibiting the scavenging effect of endothelial cells on nanoparticles, enhancing the brain capillary penetration ability of non-viral gene vectors, and realizing the targeted delivery of gene drugs to brain tumors [68]. Xiaobing et al. encapsulated the autophagy inhibitor hydroxychloroquine and the sonodynamic reagent CE6 together through the “all in one” vector for the treatment of glioma, and finally showed a good synergistic effect [69]. Amphiphilic polymers have emerged as a novel class of materials garnering significant interest in oncology. Evidence from prior research indicates that these nanocarriers, which are sensitive to stimuli, have the potential to reduce adverse effects and enhance therapeutic outcomes by swiftly adapting to shifts in the tumor microenvironment [70]. Certain investigators have harnessed the properties of amphiphilic polymers to encapsulate both autophagy inhibitors and chemotherapeutic agents, aiming at counteracting multidrug resistance (MDR) in the tumor therapy. For instance, Wuliji and colleagues developed a micellar system using an amphiphilic polymer synthesized by covalently attaching hyperbranched polyacrylhydrazone (HPAH) to doxorubicin. This system incorporated the autophagy inhibitor LY294002 for the treatment of oral squamous cell carcinoma. The inclusion of LY was found to diminish the drug resistance in HN-6 and CAL-27 cells, increasing their susceptibility to DOX [71]. Metal organic frameworks (MOFs) are recognized for their high porosity, expansive surface area, and customizable nature, positioning them as superior platforms for the drug delivery. Comprising metal ions and organic ligands, MOFs have been extensively applied in tumor therapy [72]. In a study led by Chen, a zeolite imidazole framework (ZIF-8) MOF was utilized as a pH-responsive carrier for the autophagy inhibitor 3-methyladenine (3-MA), which demonstrated high drug loading capacity [73]. The interaction between zinc ions and 2-methylimidazole (MeIM) within the acidic tumor milieu resulted in the dissolution of the MOF’s crystalline structure, facilitating the release of 3-MA. The presence of MeIM induced an alkaline shift in the pH of target cells, disrupting their homeostasis and eliciting a toxic response. 3-MA, functioning as an autophagy inhibitor, targeted the class III PI3K (Vps34)/Declin-1 complex, impeding autophagosome formation, which in turn heightened the sensitivity of HeLa cells to apoptosis [74].

### 4.3. Tumor Vaccines for Autophagy Regulation

**Figure 3:**
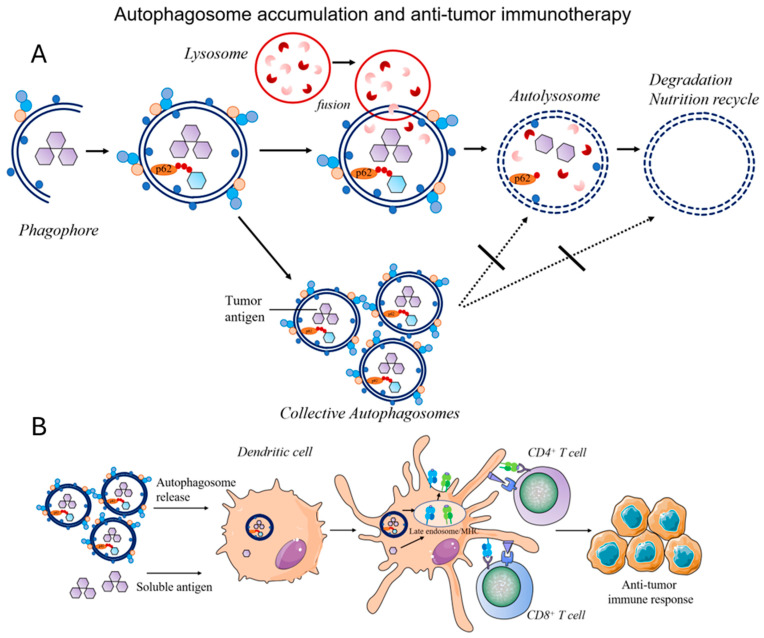
(**A**) Representation of autophagosome accumulation. (**B**) Schematic of autophagosomes for antigen presentation and activation of CD8^+^ T cells for anti-tumor immunotherapy.

Vaccination is one of the important means to prevent and treat tumors. In recent years, antigen and immune adjuvants (such as CpG (unmethylated cytosine–phosphate–guanine oligodeoxynucleotides), PD1 monoclonal antibody, etc.) were co-loaded into a nano drug delivery system, which effectively improved the immune activation effect of the vaccine [75]. Research has shown a strong relationship between tumor immunity response and autophagy [76], including inherent immunity, antigen presentation, and inhibition of immune evasion, indicating its key role in various immune responses. Some research studies have found that autophagy could strengthen MHC I presentation of HSV-1 viral antigens by autophagosomes in dendritic cells [77], or present autophagosomes as antigens for MHC II presentation [78]. Autophagy also played a critical role in cross-presentation of tumor antigens and identified autophagosomes as novel, efficient carriers for cross-presentation of tumor-associated antigens (TAA) [79]. Autophagy activators can promote dendritic cells to process and present vaccine antigens, and promote T cells to perform physiological functions. Therefore, autophagy activators can be regarded as a kind of immune adjuvant to improve the efficacy of vaccines. Yi Wang et al. modified chicken ovalbumin (OVA) and an autophagy-activating polypeptide (beclin-1) on an amino ester scaffold; thereby a nano vaccine that can activate the autophagy of dendritic cells was constructed. The system proved that when the vaccine was delivered to dendritic cells, properly activating the autophagy level could play a synergistic role and greatly enhance the immune activation effect of the vaccine [64]. These are the applications of autophagosomes as vaccines in preventing tumors. In fact, autophagosomes can also serve as therapeutic vaccines to exert anti-tumor immunotherapy effects. With the conception of tumor immunogenic cell death (ICD) proposed, some chemotherapy drugs (such as mitoxantrone and oxaliplatin) have been shown to induce tumor cell apoptosis while promoting the expression of protein molecules that cause immune attacks in the body on the surface of tumor cells, leading to anti-tumor immune responses. Therefore, numerous research on the combination of chemotherapy and immunotherapy has emerged. During chemotherapy in mice with subcutaneous colon tumors, it was found that some intact autophagosomes containing multiple tumor-associated antigens and cell death-inducing damage-associated molecular patterns (DAMPs) were released into the extracellular space and taken up by antigen-presenting cells [80]. However, autophagy activation is the first step in forming autophagosomes, which will be fused with lysosomes and gradually degraded. If autophagosomes were degraded in lysosomes, antigen presentation could be affected. Research studies have found that melanoma treated with autophagy inducers activated autophagy [81]. Further treatment with alkaline NH_4_Cl inhibited the degradation of autophagosomes, and enhanced the antigen cross-presentation, proving that autophagosomes are effective carriers for tumor antigen cross-presentation (Figure 3). Jinbo Li et al. combined an ICD inducer (SHK) with an autophagy inhibitor, hydroxychloroquine (HCQ), for colon cancer immunotherapy. HCQ enhanced SHK-induced antigen exposure of colon cancer in vitro and in vivo [82]. Similarly, Na et al. developed a thermosensitive liposome encapsulating IR780 and HCQ-Lip for cancer photothermal immunotherapy. HCQ improved the phototoxicity of IR780 and boosted IR78-induced autophagosome accumulation in breast cancer. Thereby, this strategy achieved efficient treatment of 4T1 cells by promoting the maturation of dendritic cells and the proliferation of tumor-infiltrating T cells. It also exhibited significant suppression of distant tumors due to the immune response [83]. Given that autophagy can regulate the tumor immune response, targeted autophagy can improve the efficacy of immunotherapy and overcome resistance to immunotherapy. For example, chloroquine could block autophagy-mediated MHC class I degradation, and it cooperated with dual ICB (immune checkpoint blockade) therapy (anti-PD1 and anti-CTLA4 antibodies) to produce an enhanced anti-tumor immune response in a pancreatic cancer mouse model. The use of the inhibitors SB02024 or SAR405 to inhibit VPS34 kinase activity led to increased levels of CCL5, CXCL10, and IFN-γ in the tumor microenvironment (TME), resulting in increased levels of tumor infiltration by NK cells and T cells in melanoma and colorectal cancer models [84]. In these models, VPS34 inhibition also reversed resistance to anti-PD1 or anti-PD-L1 therapy. It is believed that the nano drug delivery systems based on these strategies will receive more and more attention in the future.

## 5. Conclusions

In this review, we have delved into the application of nanomaterials and their role in regulating the process of autophagy, particularly within drug delivery systems. Autophagy, a lysosomal self-degradation pathway, is essential for maintaining cellular homeostasis. The advancement of nanotechnology has provided new perspectives and tools for autophagy research and therapy. It has been demonstrated that various nanomaterials can modulate intracellular autophagy levels, representing a novel cellular response mechanism for disease treatment. The application of nanomaterials in autophagy regulation primarily focuses on several aspects: firstly, as autophagy probes, leveraging the unique physical and chemical properties of nanomaterials to enhance the detection and quantification of autophagy activity within cells; secondly, as autophagy modulators, certain nanomaterials can induce or inhibit autophagy based on the desired therapeutic outcomes; and lastly, as drug delivery vehicles, nanocarriers can improve the targeting, bioavailability, and efficacy of drugs while reducing systemic toxic side effects. Despite the immense potential of nanomaterials in autophagy regulation, there are challenges and risks. For instance, the interaction of nanomaterials with cellular organelles may lead to cellular damage, and the balance between damaging and protective effects of autophagy induced by nanomaterials is critical in their design and therapeutic application. Future research should aim to optimize the beneficial effects of nanomaterial-induced autophagy while minimizing potential harm.

## Figures and Tables

**Table 1:** Bioactive nanomaterials for autophagy regulation.

Effect on Autophagy Modulation	Nanomaterials	Cells and Models
Autophagy inhibition and lysosomal dysfunction	Gold nanoparticles [48]	NRK
	Graphene oxide nanocolloids [49]	Mouse embryonic stem cells
	Graphene oxide QDs [50]	GC-2; TM4
	Silver nanoparticles [51]	Primary MEF; Hela; THP-1 monocytes
	Silica nanoparticles [52]	L-02
Autophagy induction and lysosomal activation	Single-walled carbon nanotubes [53]	Primary glia from CRND8 AD, murine
	Graphene oxide nanoparticles [54]	HeLa
	Iron oxide nanoparticles [35]	A549; IMR-90
	Titanium dioxide nanoparticles [39]	HaCaT
	Bismuth nanoparticles [44]	HEK293
	Tetrahedral DNA nanostructures [55]	Chondrocytes
Autophagy-mediated chemosensitization	Fullerene C60 [56]	HeLa; MEF; MCF-7
	CdTe and CdTe/CdS/ZnS QDs [57]	PC12; HEK293

## Data Availability

Data are contained within the article.
